# The development of probiotics and prebiotics therapy to ulcerative colitis: a therapy that has gained considerable momentum

**DOI:** 10.1186/s12964-024-01611-z

**Published:** 2024-05-14

**Authors:** Jing Guo, Liping Li, Yue Cai, Yongbo Kang

**Affiliations:** 1 Department of microbiology and immunology, School of Basic Medical Sciences, Shanxi Medical University, Taiyuan, Shanxi China; 2 Faculty of Life science and Technology, Kunming University of Science and Technology, Kunming, Yunnan China

**Keywords:** Ulcerative colitis, Probiotics, Prebiotics, Treatment, Gut microflora, Inflammation, Mechanism

## Abstract

Ulcerative colitis (UC) is increasingly common, and it is gradually become a kind of global epidemic. UC is a type of inflammatory bowel disease (IBD), and it is a lifetime recurrent disease. UC as a common disease has become a financial burden for many people and has the potential to develop into cancer if not prevented or treated. There are multiple factors such as genetic factors, host immune system disorders, and environmental factors to cause UC. A growing body of research have suggested that intestinal microbiota as an environmental factor play an important role in the occurrence and development of UC. Meanwhile, evidence to date suggests that manipulating the gut microbiome may represent effective treatment for the prevention or management of UC. In addition, the main clinical drugs to treat UC are amino salicylate and corticosteroid. These clinical drugs always have some side effects and low success rate when treating patients with UC. Therefore, there is an urgent need for safe and efficient methods to treat UC. Based on this, probiotics and prebiotics may be a valuable treatment for UC. In order to promote the wide clinical application of probiotics and prebiotics in the treatment of UC. This review aims to summarize the recent literature as an aid to better understanding how the probiotics and prebiotics contributes to UC while evaluating and prospecting the therapeutic effect of the probiotics and prebiotics in the treatment of UC based on previous publications.

## Introduction

Ulcerative colitis (UC) is a chronic non-specific intestinal inflammatory disease [1]. UC becomes an important health problem, because it’s high morbidity. Especially in newly industrialized countries [2]. Research shows that the incidence of UC is 10 to 20 patients per 100,000 people every year [3]. UC often presents with recurrent attacks. And the inflammatory of UC will become a factor of colon cancer in the long run [4]. The pathogenic factors of UC are sophisticated, it is related to intestinal microbiota, immune function of the body (For example, UC is closely related with Th2 cells) [5], genetic factor and environment factor (e.g. life-style, dietary habits) and so on [6]. wherein, intestinal microbiota is one of the most important factor that arise UC [7]. Therefore, we can use probiotics to regulate the intestinal flora in the treatment of UC [8, 9]. A growing body of research has shown that probiotics and prebiotics can bring about remission the symptoms of UC improving intestinal mucosal homeostasis, ameliorating the intestinal microbiota environment, regulating the body’s immune function. Therefore, probiotics and prebiotics may be a very safe and efficient treatment for UC. At the same time, it can greatly reduce the financial burden of patients. Furthermore, New techniques have made it possible to attempt systematic studies of probiotics prebiotics, which can provide more specific information about their functions and pathological variations. This review summarizes cutting-edge research on probiotics and prebiotics treatment for UC, existing issues in probiotics treatment and prebiotics therapy, the future of probiotics and prebiotics, and microbial therapeutics.

## Pathogenesis of UC

Ulcerative colitis is a chronic inflammatory disorder of the gastrointestinal tract. It is characterized by a progressive decline in health. UC is marked by inflammation of the mucosal lining, usually confined to the colon and rectum [10]. The pathogenesis of UC is closely related to a variety of factors, such as genetics and environment [11]. Statistically, genetics can only explain 7.5% of the variation in disease and has little predictive power for phenotype. Therefore, it has limited clinical application. Examples of loci associated with increased susceptibility to UC including genes associated with barrier function and human leukocyte antigen, such as HNF4A and CDH1 [12, 13]. Environment plays an important role in the development of UC. Such as, living condition, hygiene, diet, etc. While UC is mainly due to immune dysfunction and intestinal barrier dysfunction. Colonic epithelial cells (colonocytes), as the first line of defense of the gut immune system, are closely related to the pathogenesis of the UC. Research findings, the expression of peroxisome proliferator-activated receptor γ (PPAR γ) is reduced in the colonocytes in patients with UC. And the reduced expression of PPAR γ, which is a nuclear receptor that downregulates inflammation, will stimulate an inflammatory cascade responses through a series of immune responses, leading to the production of large quantities of inflammatory factors [14]. Also, when certain genes in the intestinal epithelium are functionally deficient, it may lead to disruption of the intestinal barrier function [10]. The deficiency or malfunction of various immune cells and the abnormal expression of cytokines, which play an important signaling function, can also lead to inflammation, which, if prolonged, can lead to the development of UC. The intestinal immune system also involves the intrinsic and adaptive immunity [15], involving a variety of immune cells and molecules and others. If dendritic cells abundantly express Toll-like receptors (TLR) which can recognize pathogen pattern receptors, this will leads to the activation of several inflammatory signaling pathway, such as NF-κB [16] and MAPK pathway, triggering an inflammatory response. The production of large amounts of pro-inflammatory factors affects the differentiation of immune cells such as T cell differentiation towards subpopulation. For example, massive activation of Th2 cells leads to high expression of IL-13, which induces apoptosis of epithelial cells and disrupts the integrity of mucosal barrier [17, 18]. Other T helper cells also play an important role in UC. And some research suggest that Breg deficiency may also associated with UC [19]. The damage of the intestinal mucosal barrier is also an important causative factor in UC. Intestinal secretory dysfunction such as decreased secretion of antimicrobial peptides and mucus layer, or structural defects of intestinal barrier including occludin, ZO-1, ZO-2 and so on. It has been found that the disruption of human gut microbiota, the largest collection of microbes within the body [20], is critical in the progression of UC, but the specific mechanism is not yet clear.

## The role of gut microflora in UC

**Fig. 1:**
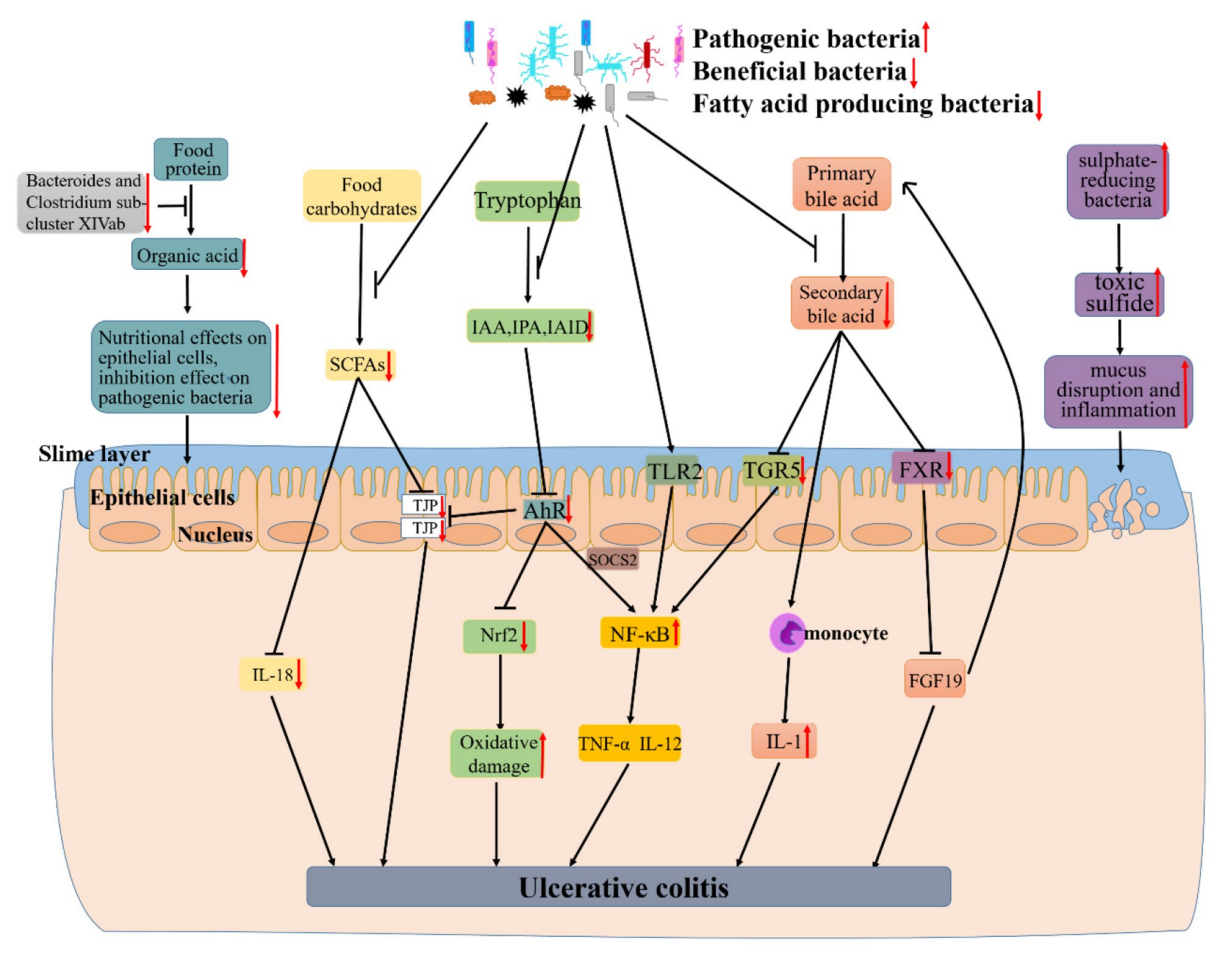
The mechanism of UC caused by dysbiosis of gut microbiota. Research findings, the decline of certain beneficial bacteria inhibits the conversion of food protein into organic acid which can nourish epithelial cells and inhibit pathogenic bacteria. Firmicutes as a major producer of butyrate (a kind of SCFAs), its decline leads to lower intestinal SCFAs. Leading the decreased secretion of epithelial repair cytokine interleukin-18, reduced the integrity of epithelial cells, and inhibited goblet cells secrete mucin and modification of tight junctions. And the decline of some gut microbiota also can lead to a decrease of indoles and their derivatives (e.g., IAA, IPA and IAID) which is produced by tryptophan. Thereby reducing the activation of AhR, a member of the activation of PER-ARNT-SIM (PAS) superfamily of transcription factors. The activation of AhR can inhibited the expression of NF-κB in a manner dependent on suppressor of cytokine signaling 2 (SOCS2). And AhR can also maintains the integrity of intestinal barrier activation by increasing the expressions of intestinal tight junction protein (TJPs) or activating the AhR-Nrf2 pathway. All of these effects were reversed due to the decrease of IAA, IPA or IAID. Thus lead to the increase of inflammatory factors (e.g., TNF-α and IL-17) and oxidative damage. Other researchers found that certain pathogenic bacteria such as Bacteroides (B.) fragilis and capsular lipopolysaccharide A can activate NF-κB signaling pathway and promote the secretion of inflammatory factors. The gut microbiota dysbiosis can also lead to the decreased synthesis of secondary bile acid. And secondary acid act as high-affinity ligands for TGR5 and FXR, its decline can promote NF-κB activation to synthesize inflammatory and the expression of proinflammatory cytokines secreted by monocyte and downregulate the expression of FGF19 and promote the synthesis of bile acids thus increasing its toxicity effect on tissues. As an intestinal pathogen, the increase of sulphate-reducing bacteria leads to cell disintegration and inflammatory via toxic sulfide. All of these can lead to the occurrence and development of UC.

Gut microflora lives on intestinal mucosal and forms bacterial layer. Thus, there is a strong and complex relationship between gut microbiota and gut. Intestinal dysbacteriosis can leads to a decrease in intestinal defense function and immune regulatory function. Furthermore, the decrease of the body immune function and an increase in associated pathogenic factors leading to the intestinal mucosal invasion or exacerbates the gastrointestinal diseases [6]. Recently, a large number of studies have shown that alterations of intestinal microbiota can play an important role in the occurrence and development of UC. Meanwhile, some studies have shed light on UC subjects exhibiting alterations in the relative abundance of “beneficial” and potentially “harmful” bacteria compared to healthy subjects. The existence of a link between UC and the gut microbiota was indicated based on studies in animals and patients with UC. Changes of gut microbiota together with their-derived products and metabolites account for the important factors to promote UC occurrence. Here, the possible mechanisms of microbiome-gut action in promoting UC occurrence are discussed as well as outlined in Fig. 1.

A large number of studies have shown that patients with UC have a decrease in the bacterial diversity of gut microbiota [21]. Animal study results indicate a close association between gut microbiota and UC. Li et al. found that Firmicutes and Proteobacteria increased, whereas Bacteroidetes decreased in UC rats. And *Lactobacillus*, *Lachnospiraceae_NK4A136_group*, *Prevotella_9* and *Bacteroides* were dominant genera in the model group [22]. Consistent with animal studies, the existence of a link between UC and the gut microbiota was indicated based on studies in patients with UC. Guo et al. also found that the abundance of *Bacteroides* and *Clostridium sub-cluster XIVab* as well as the concentration of organic acids significantly decrease by comparing with healthy individuals [23]. Similarly, Mizoguchi et al. shown that UC patients harbored relatively more abundant Actinobacteria, Proteobacteria and Tenericutes [24]. A comparison between UC and healthy individuals differed in the composition and diversity of the microbiota, with an upward trend in the *Clostridium cluster IX* and a decreased *Clostridium cluster XIVa* in patients with UC [25]. Consistent with the above results, there is a reduced amounts of bacterial groups from the *Clostridium cluster XIVa*, and the levels of Bacteroidetes was increased [26].

In addition, Kotlowski et al. found that the numbers of *Escherichia coli* were high in the rectal tissue of patients with UC [27]. By comparing with healthy controls, Xu et al. showed that the inflamed mucosa had more Proteobacteria (e.g. *Escherichia–Shigella*) and fewer Firmicutes (e.g., *Enterococcus*) [28]. As demonstrated by Schwiertz et al., Patients with active UC have lower cell counts of *Bifidobacterium* than healthy controls [29]. Another study found that the sulfate-reducing bacteria which is the dominant microflora in UC, it may proliferate with the release of toxic sulfide [30].

Recently, Verma et al. shown that during the active and remission stages of UC cases, the proportions of *Bacteroides*, *Eubacterium*, and *Lactobacillus* spp. are decrease [31]. Similarly, in another analysis of mucosa-associated flora in UC patients, it was learned that UC patients contained proportionally less *Firmicutes*, and correspondingly more *Bacteroidetes* [32]. Tahara et al. demonstrated that *Fusobacterium nucleatum* is common which is isolate from human intestinal biopsy from UC, compared to healthy controls [33].

In keeping with these results, Machiels et al. found that there is a decrease of the *Roseburia hominis* and *Faecalibacterium prausnitzii* in patients with UC [34]. Lepage et al. demonstrated that patients with UC are characterized by more Actinobacteria and Proteobacteria and less bacteria from the Lachnospiraceae and Ruminococcaceae families [35]. Likewise, a significant reduction was found on the UC mucosa compared with the non-IBD controls, that is levels of *Clostridium clostridioforme*, the *Eubacterium rectale* group, *Faecalibacterium prausnitzii*, *Bifidobacteria*, *Lactobacilli*, and *Clostridium butyricum* [36]. Consistent with the above results of this study, patients with UC in remission compared to that of controls, there is a loss of *Bacteroides*, *Escherichia*, *Eubacterium*, *Lactobacillus*, and *Ruminococcus* spp [37].

Recently, Hu et al. [38] found that the decreased of the dominant bacteria that digest food carbohydrates to short chain fatty acid (SCFA) lead to the reduce of intestinal barrier integrity (for example, the decrease of TJPs in colon). Guo et al. [23] also found that SCFAs can affect the secretion of the epithelial repair cytokine interleukin-18. And they found that the decreased of *Bacteroides* and *Clostridium sub-cluster XIVab* leading to the decrease of organic acid, which reduces the trophic effect of organic acid to epithelial cells and the inhibitory effect on pathogenic bacteria [39]. Agus et al. [40] found that the reduced of certain intestinal flora inhibited the conversion of tryptophan to indole and its derivatives, and AhR as a receptor of indole and its derivatives, its activation will reduced, thereby inhibiting the intestinal TJP and AhR-Nrf2 pathway, leading to the reduced of intestinal barrier integrity and increased oxidative stress [41]. Rothhammer et al. [42] demonstrated that the reduce of AhR can promote the activation of NF-κB pathway in a manner dependent on suppressor of cytokine signaling 2 (SOCS2), then increase the expression of a number of inflammatory factors, including TNF-α and IL-12 et al. It is reported that some bacteria regulate the secretion of TNF-α and IL-12 by activating the NF-κB pathway through TLR2 receptor [43]. Iracheta et al. [44] found that primary bile acid are converted to secondary bile acid by gut microorganisms after being secreted into gut through a series of reactions, and that a decline of these gut microorganisms leads to a decrease of secondary bile acid. The decrease of secondary bile acid, which act as high-affinity ligands for TGR5 and FXR, leads to a decreased activation of TGF5 and FXR. The inhibitory effect of TGR5 on NF-κB is reduced, thereby promoting the activation of NF-κB. Reduced activation of FXR down-regulates the expression of FGF19, then its inhibitory effect to hepatic bile acid is declined, leading to a further increase of bile acid and exacerbating the development of inflammation [45]. And the decrease of secondary bile acids promote the secretion of pro-inflammatory factors by monocytes [46]. Figliuolo et al. [47] found that the increase of sulphate-reducing bacteria lead to an increase of toxic sulfide, which cause the disruption of gut epithelial cell and increase intestinal inflammatory.

Taken together, these results provide further insights into a role for gut microbiota in the pathogenesis of UC and might potentially serve as guidance for the interventions of UC by manipulating gut microbiota.

## Research advances existing challenges IBD treatment

At present, there are many various treatment methods for IBD. Conventional treatment is the use of pharmacotherapy, including aminosalicylates, corticosteroids (CSs), immunomodulators (e.g., thiopurines (TPs), methotrexate (MTX), and calcineurin inhibitors), and biologics (e.g., pro-inflammatory cytokine inhibitors and integrin antagonists). Surgical resection and other methods including apheresis therapy, antibiotics, probiotics and prebiotics can also be used for treatment [48]. However, the side effects and high reccurence rate of these substances and methods limit there application. For example, research found, although aminosalicylates have been used in the treatment of IBD for the past 80 years, its efficacy remains controversial. And its mild side effects include diarrhea, nausea, abdominal pain, flatulence and others [49]. Severe cases can lead to infertility and anemia. CSs inhibits the transcription of certain inflammatory factors [50] and regulate the expression of certain anti-inflammatory genes [51] through certain signaling pathways. And it has many side effects, including diabetes mellitus, hypertension, venous thromboembolism (VTE), etc [52].. Some patients may also have dependence on this medication [53]. TPs inhibits intestinal inflammatory response by regulating T cell proliferation and activation. But TPs can cause side effects such as liver damage [54] and gastrointestinal intolerance [55]. MTS excerts its effects also by downregulating inflammatory factors. But it can cause adverse reactions such as fatigue, diarrhea, pneumonia and rash [51]. Calcineurin inhibitors also supresses inflammatory responses by interfering with signaling pathways. The incidence of side effects of calcineurin inhibitors is high, including renal function damage, hyperkalemia and infectious diseases and so on [56]. Anti-TNF therapy will inhibit the secretion of pro-inflammatory factor TNF-α. Anti-IL-12/23 therapy works by inhibiting the production of pro-inflammatory factor IL-12 and IL-23 by antigen-presenting cells. Anti-integrin therapy inhibits the accumulation of white blood cells in intestinal and alleviates intestinal inflammatory. But these biological agents are expensive and many patients may experience unresponsive and intolerant states. Therefore, it is urgent to study effective and safe methods to treat UC.

In the recent years, regulating gut microbiota has become a hot topic in the treatment of UC. Therefore, as a promising method for treating IBD, probiotics act as live microorganisms have therapeutic effects on IBD which is caused by intestinal ecological disorders and other reasons. The treatment of IBD can be achieved through its antioxidant effects [57], the regulatory effect on gut microbiota [58], anti-inflammatory effect [59], the promotion effect to intestinal barrier integrity [60] and so on. As an indigestible food ingredient, prebiotics can also be used to treat or alleviate UC by regulating the redox system, immune system, etc. It can also selectively regulate colon microbiota, for example, enhancement of beneficial intestinal bacteria and inhibition of the growth of pathogenic microorganisms. All of these suggests that probiotics and prebiotics have a lot of room to develop as new form of treatment.

## Effect and mechanism of probiotics and prebiotics in treating UC

**Fig. 2:**
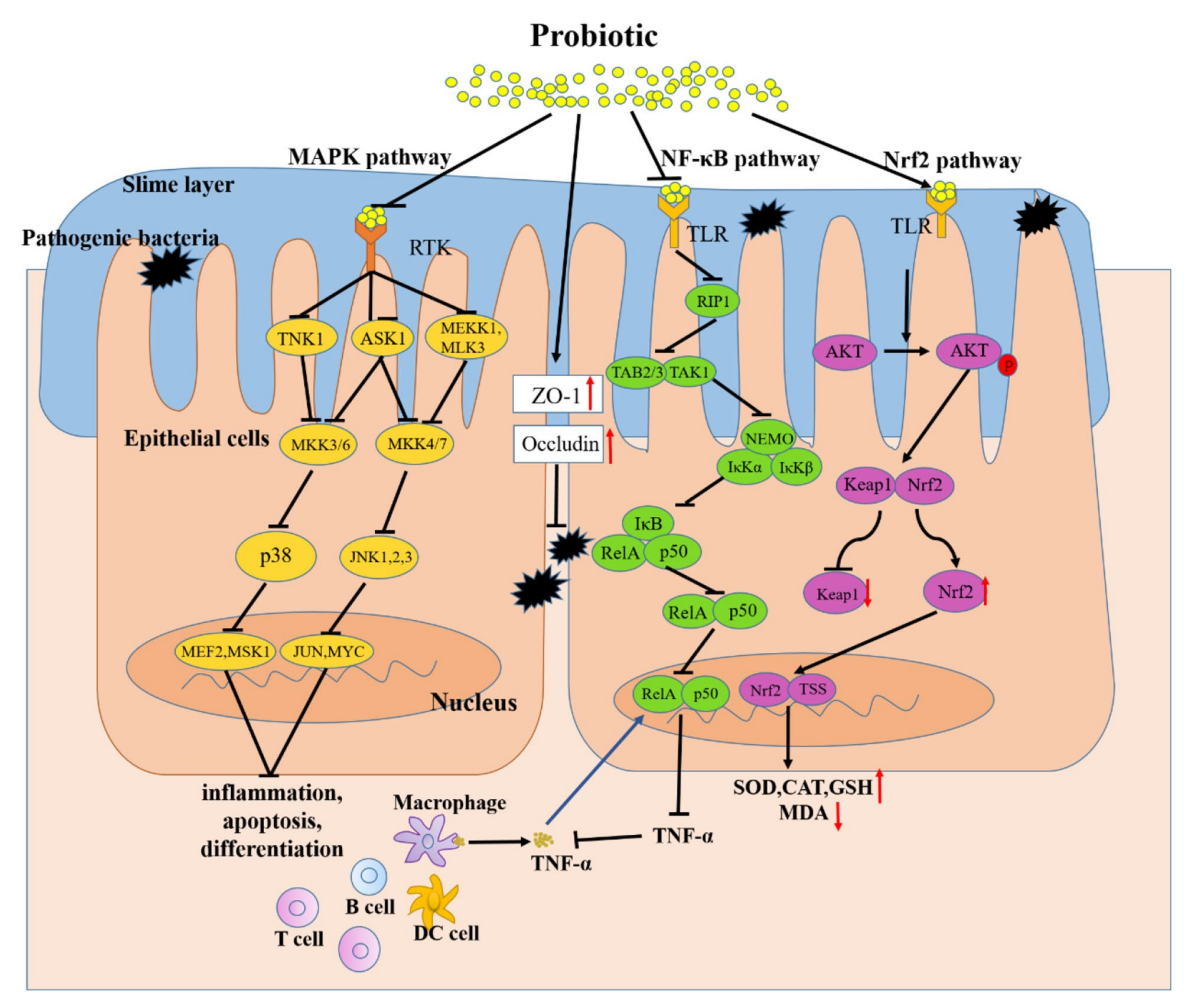
The potential mechanism of probiotics in alleviating Ulcerative Colitis (UC). Probiotics that enter the gut can bind with corresponding receptors (e.g. PTK) which are on the intestinal epithelial cells, then inhibit its stimulation to MAPKKK (e.g. TNK1, ASK1, MEKK1, MLK3), further suppress the activation of MAPKK (e.g. MKK3/6, MKK4/7) which are activated by MAPKKK, thereby inhibiting the activation of MAPK (e.g. p38, JNK1,2,3). Blocking the transcription factor transcribe of relevant genes (e.g. Cyclin D1, Raf). Finally, inhibition the inflammatory, apoptosis, and differentiation activated by this pathway. Meanwhile, probiotics protect the intestinal barrier by increasing the levels of tight junction proteins of ZO-1 and Occludin between intestinal epithelial cells, preventing the invasion of pathogenic microorganisms. In addition, probiotics can bind with its receptors (e.g. TLR) on the intestinal epithelial cells, inhibiting the activation of adaptor protein (e.g. RIP1) and suppressing the recruitment of TAB/TAK complex, thereby inhibiting the ubiquitination degradation of IκB by ubiquitinatingNEMO. Prevents the release of NF-κB proteins (RelA/p50) to nucleus. Ultimately inhibits the transcription of proinflammatory factors (e.g. TNF-β) and reduces the promotion effect of TNFα releasing by macrophages to this pathway. Meanwhile, probiotics act on intestinal epithelial cells-associated receptors (e.g. TLR), then phosphorylate AKT, and inhibit the degradation of Nrf2. Nrf2 enters the nucleus and promotes the expression of a range of cytoprotective genes (e.g. SOD, CAT, GSH).

Probiotics are nonpathogenic living microorganisms which, when administered in adequate amounts, have been shown to confer health benefits to the host and regulate intestinal microecological balance. Probiotics are widely used in medical application to prevent or treat many diseases, such as obesity [61], hepatocellular Carcinoma [62], autoimmune hepatitis [63], diabetic retinopathy [64], and alcoholic liver disease [65] and so on. The therapeutic effects of probiotics on UC have also been confirmed in animals and humans (Tables 1 and 2). Thus, therapeutic interventions with probiotics may offer new treatment for UC. Here, the possible effects and mechanisms of probiotics in the treatment of UC are summarized in Fig. 2.

**Table 1:** Changes in microbiota composition in animals associated with UC and probiotics therapeutic strategies CCMN, *Companilactobacillus crustorum* MN047; Hao9, *Lacticaseibacillus rhamnosus* Hao9; *L.p* R3, *Lactobacillus paracasei* R3; TPM and probiotics, TPM and *Lactobacillus animalis*-BA12, *Lactobacillus bulgaricus*-LB42, *Lactobacillus paracasei*-LC86, *Lactobacillus casei*-LC89 and *Lactobacillus plantarum*-LP90; ZS40, *Lactobacillus fermentum* ZS40.

Models	Disease	Implicated microbiota	New therapeutic	Implicated microbiota	Reference
Mice	DSS-induced UC	*Acinetobacter*↑, *Bacteroides*↑, *Coprobacillus*↑, *Alistipes*↓, *Bifidobacterium*↓, *Blautia*↓	*B.longum* CCFM1206	*Alistipes*↑, *Bifidobacterium*↑, *Blautia*↑, *Acinetobacter*↓, *Lachnospiraceae* A2↓	[66]
Mice	DSS-induced UC	*B.infantis*↓, *Faecalibacterium*↓, *Lactobacillus*↓	*B. infantis*	*B. infantis*↑	[67]
Mice	DSS-induced UC	No	*B.xylanis olvens*AY11-1	*Blautia spp*↑, *ellaceae* UCG- 001↑	[68]
Mice	DSS-induced UC	*Intestinimonas*↑, Parabacteroides↑, *Parasutterella*↑, *Akkermansia*↓, Alistipes↓	CCMN	*Bacteroidaceae*↑, *Burkholderiace*ae↑, *Akkermansiaceae*↓, *Eggerthellaceae*↓	[69]
Mice	DSS-induced UC	*Akkermansiaceae*↑, *Bacteroidaceae*↑, *Lachnospiraceae*↓, *Lactobacillaceae*↓	*Se-B. longum* DD98	*Firmicutes*↑, *Bacteroidetes*↓	[70]
Mice	DSS-induced UC	*Bacteroides*↑, *Turicibacter*↑, *Lactobacillus*↓, *Ligilactobacillus*↓, *Romboutsia*↓	Hao9	*Faecalibaculum*↑, *Romboutsia*↑	[71]
Mice	DSS-induced UC	No	*L.p* R3	No	[72]
Mice	DSS-induced UC	*Firmicutes*↑, *Patescibacteria*↑, *Bacteroidota*↓, *Actinobacteriota*↓	*S.boulardii* and the postbiotics	*Turcibacter*↑	[73]
Mice	DSS-induced UC	No	*L. lactis*	No	[74]
Mice	Acetic acid induced UC	No	*L. acidophilus*	No	[75]
Mice	DSS-induced UC	No	*S. boulardii*	No	[76]
Mice	DSS,-induced UC	No	*E. faecium*	*Butyricicoccus* sp.↑, *Lactobacillus* sp.↑, *Bifidobacterium* sp.↑, *Ochrobactrum* sp.↓, *Acinetobacter* sp.↓	[77]
Mice	DSS-induced UC	*Lachnospiraceae*↑, *Akkermansia*↑, *Muribaculaceae*↓, *Alistipes*↓, *Ligilactobacillus*↓, *Lactobacillus*↓	TPM and probiotics	*Muribaculaceae*↑, *Alistipes*↑, *Ligilactobacillus*↑, *Lactobacillus*↑	[78]
Mice	DSS-induced UC	*Proteobacteria*↑, *Actinobacteria*↓, *Bacteroidota*↓	*B. bifidum* H3-R2	*Bifidobacterium*↑, *Lactobacillus*↑, *Enterobacter*↓, *Enterococcus*↓, *Streptococcus*↓	[79]
Mice	DSS-induced UC	No	ZS40	No	[80]

**Table 2:** Changes in microbiota composition in human associated with UC and probiotics therapeutic strategies

Models	Disease	Implicated microbiota	New therapeutic	Implicated microbiota	Reference
Human	UC	No	*L.delbruekii* and *L.fermentum*	No	[82]
Human	UC	No	*L.salivarius, L.acidophilus and B.bifidus strain* BGN4	No	[83]
Human	UC	No	Symprove	No	[84]
Human	UC	No	BIO-THREE	bifidobacteria↑	[85]
Human	UC	No	probiotic capsules	No	[86]
Human	UC	No	*B.infantis* 35,624	No	[87]
Human	UC	No	AKK	No	[88]
Human	UC	No	*Lactobacillus casei* Zhang, *L.plantarum* P-8 and *B.animalis* subsp. *lactis* V9	*Eubacterium*↑, *Pediococcus*↑, *Weissella*↑, *Lactococcus*↓, *Meiothermus*↓	[89]

Symprove, *Lactobacillus rhamnosus* NCIMB 30,174, *Lactobacillus plantarum* NCIMB 30,173, *Lactobacillus acidophilus* NCIMB 30,175 and *Enterococcus faecium* NCIMB 30,176; BIO-THREE, *Streptococcus faecalis* T-110, *Clostridium butyricum* TO-A and *Bacillus mesentericus* TO-A; probiotic capsules, *Lactobacillus paracasei* (A234), *Lactobacillus gasseri* (A237), *Lactobacillus rhamnosus* (A119), *Lactobacillus rhamnosus* (A193), *Lactobacillus acidophilus* (A118), *Lactobacillus plantarum* (A138), *Lactobacillus casei* (A179), *Lactobacillus reuteri* (A113), *Lactococcus lactis* (A328), *Bifidobacterium animalis* subsp.*lactis* (A026), *Bifidobacterium breve* (A055), *Bifidobacterium longum* subsp.*Longum* (A027), *Bifidobacterium bifidum* (A058), *Bifidobacterium longum* subsp.*Infantis* species (A041); AKK, *Akkermansia muciniphila*

### Probiotics therapy

#### Experimental studies

Convincing evidence from animal studies indicate that probiotics treatment can relieve UC (Table 1). Wu et al. [66] found that the use of *Bifidobacterium longum* CCFM1206 to treat Dextran-Sulfate-Sodium (DSS) induced Colitis mice will promotes the conversion of Glucoraphanin (GRP) to sulforaphane (SFN). SFN help to upregulate the Nrf2 signaling pathway and inhibit the NF-κB activity, which can ameliorate DSS-induced colitis. The result also indicated that the intervention of *B.longum* CCFM1206 could relieve the dysbiosis of intestinal microbiota. That is, promoted the proportion of *Alistipes*, *Bifidobacterium*, *Blautia* and *Lachnospiraceae* NK4A136 group and inhibited the proportion of *Acinetobacter*, and Lachnospiraceae A2 in the gut. Similar study, Han et al. [67] demonstrated that *Bifidobacterium infantis* enhances genetic stability by maintaining the balance of gut flora to increase anaphase-promoting complex subunit 7 (APC7) expression in colonic tissues, changing gut flora such as an increase in *B.infantis*. Then reducing DSS-induced colonic inflammation. Consistent with the above results, Fu et al. [68] found that *Bacteroides xylanisolvens* AY11-1 regulate the intestinal microbiota through the efficient degradation of alginate, improving the dysbiosis of intestinal ecology and promoting the growth of beneficial bacteria, for example, the increase of *Blautia spp* and *Prevotellaceae* UCG-001. Then ameliorated the symptoms of DSS-induced UC in mice. Wang et al. [69] revealed that the administration of probiotic *Companilactobacillus crustorum* MN047 in DSS-induced UC mice resulted in the expression of tight junctions, and down-regulation of pro-inflammatory and chemokine expression. It was also found that an increase of goblet cells, MUCs, TFF3, and TJs in the probiotic group, which demonstrated that the treat with CCMN could enhance the gut barrier function. And confirmed by fecal microbiota transplantation (FMT), the mechanisms of CCMN alleviating UC were partly due to its modulation to gut microbiota. The result showed that an increase in *Bacteroidaceae* and *Burkholderiaceae* and a decrease in *Akkermansiaceae* and *Eggerthellaceae*. Hu et al. [70] also found that Selenium-enriched *Bifidobacterium longum* DD98 administration alleviated the symptoms caused by DSS, inhibited the expression of the pro-inflammatory cytokines, decreased the level of oxidative stress, promoted the expression of tight junction proteins, inhibited the activation of toll-like receptor 4 (TLR4), and regulated the gut flora. They found that after the treatment of *Se-B. longum* DD98, the phylum of *Bacteroidetes* decreased and the phylum of *Firmicutes* increased. All of the above can be effective attenuated DSS-induced colitis in mice. In another study, the results of Han et al.’s [71] study of *Lacticaseibacillus rhamnosus* Hao9 in DSS-induced UC mice showed that the use of Hao9 attenuated weight loss which is caused by DSS, lowered DAI scores, attenuated colonic damage and inflammatory infiltrates and promoted the growth of *Faecalibaculum* and *Romboutsia* in the gut. The researcher attributed the observed effects of Hao9 on UC to its ability to inhibit lipopolysaccharide-induced intestinal IκB activation of mice. Consistent with the above results, Huang et al. [72] also showed that *Lactobacillus paracasei* R3 supplementation improved the general symptoms of murine colitis, attenuated inflammatory cell infiltration and more. And it was showed that the imbalance of Treg/Th17 cell in the intestinal inflammation caused by DSS was restored after treatment with *L.p* R3. Similarly, Xu et al. [73] investigated the effect of *Saccharomyces boulardii* and its postbiotics on DSS-induced UC in mice, showing that both *S. boulardii* elements and its postbiotics could significantly alleviate weight loss, reduce colonic tissue damage, regulate the balance of pro/anti-inflammatory cytokines in serum and colon, promote the expression of colonic tight junction proteins, and regulate the stability of intestinal microecology in mice. Changing in the bacterial flora were characterized by a significant increase in *Turcibacter* at the genus level, which collectively attenuate DSS-induced colitis. Komaki et al. [74] administered *Lactococcus lactis subsp.lactis* JCM5850 to mices with colitis induced by DSS and found that moderate amounts of *L. lactis* had a mitigating effect on colitis. In keeping with these results, Hizay et al. [75] also found that *Lactobacillus acidophilus* reduces abnormally high levels of serotonin in colon tissue in acetic acid-induced UC and relieves inflammation in intestinal tissue. As with the results above, Gao et al. [76] made *Saccharomyces boulardii* into suspension, observing its effect on DSS induced colitis in mice. The results suggested that *S. boulardii* can alleviate the clinical symotoms of colitis in mice exposed to DSS and the histological lesions. And it was found that the mechanism of *S. boulardii* to treat UC is inhibite nuclear transcription factor kappa B (NF-κB) and activate nuclear factor erythroid 2-related factor 2 (Nrf2) signaling pathway. As demonstrated by He [77] et al., *Enterococcus faecium* administration prevented DSS-induced intestinal inflammation and intestinal flora dysbiosis and particially repaired the damage to intestinal mucosal barrier and tight junctions. The modulatory effect on intestinal flora was characterized by an increase in *Butyricicoccus* sp., *Lactobacillus* sp., and *Bifidobacterium* sp. and a decrease in *Ochrobactrum* sp. and *Acinetobacter sp.*.

By studying the effects of tetrapeptide from maize (TPM) and probiotic (5 *Lactobacillus* strains: *L.animalis-*BA12, *L.bulgaricus-*LB42, *L.paracasei-*LC86, *L.casei-*LC89 and *L.plantarum-*LP90) in mice with DSS-induced UC, Li et al. [78] found that it could reduce the level of oxidative stress, attenuate the loss of kidney and colon, and regulate the intestinal flora to alleviate the inflammatory effects of UC. Wherein, the modulation effect to gut microbiota in manifested as an increase in *Muribaculaceae, Alistipes, Ligilactobacillus* and *Lactobacillus*. Recently, Shang et al. [79] reported that *Bifidobacterium bifidum* H3-R2 can effectively alleviate of pathogenesis by inhibiting inflammatory signaling, maintaining intestinal ecological homeostasis, and protecting colonic integrity. *B.bifidum* H3-R2 administration similarly affected the composition of gut microbiota, showing that *B.bifidum* H3-R2 caused a significant increase in the abundance of *Bifidobacterium* and *Lactobacillus* and a decrease in *Enterobacter*, *Enterococcus* and *Streptococcus*. Chen et al. [80] also discovered *Lactobacillus fermentum* ZS40 could inhibit DSS-induced mice colon shortening, colon damage, and intestinal wall thickening. It does so by inhibiting the activation of NF-κB and MAPK signaling pathways, and ultimately relieved inflammation.

To sum up, these results provide important clues for the design and use of more effective probiotic agents to treat UC and may provide new insights into the mechanisms by which host-microbe interactions confer the protective effect. And probiotics as additionally supplemented active micro-organisms, may have better value in clinical applications as drugs in the future [81].

#### Clinical studies

There are many contributing factors to UC, but much evidence suggests a strong link between host gut microbes and the treatment of UC pathogenesis, and suggests that mediation of gut microbes is the key to treating UC. Probiotics have been shown to alleviate UC by altering the composition of the gut microbiota and many other ways. A growing number of clinical trials have also demonstrated the therapeutic effects of probiotics in UC (Table 2). As early as 2010, Hegazy et al.’s [82] study showed that administration of probiotics (*Lactobacillus delbruekii* and *L. fermentum*) not only decreased the NF-κB DNA binding activity, but also reduced the accumulation of leukocytes, and down-regulated levels of pro-inflammatory factors, and thereby ameliorated the severity of the colitis. Similarly, in order to study the long-term effect of probiotics on UC, Palumbo et al. [83] conducted a clinical study and the results of the study showed that patients in the probiotics (*L. salivarius*, *L.acidophilus* and *B.bifidus strain* BGN4) treatment group had better outcomes which is reflected through MMDAI. Thus, the use of probiotics may enhance the anti-inflammatory effect. Similar results, Bjarnason et al. [84] tried to prove the impact of the probiotic Symprove (including *Lactobacillus rhamnosus* NCIMB 30,174, *Lactobacillus plantarum* NCIMB 30,173, *Lactobacillus acidophilus* NCIMB 30,175 and *Enterococcus faecium* NCIMB 30,176) which contains four naturally occurring bacterial strain for this experiment. Research showed that Symprove are associated with reduced intestinal inflammation in UC patients. In line with these results, Tsuda et al. [85] gave patients with moderate to severe UC treated with BIO-THREE (containing *Streptococcus faecalisa* T-110, *Clostridium butyricum* TO-A and *Bacillus mesentericus* TO-A). Researchers found that the treated with BIO-THREE were able to improve clinical and endoscopic examinations in about half of UC patients who were intolerant to conventional therapy. And its intake improved intestinal microflora, the main change may be an increase in bifidobacteria. After a six-week study, Agraib et al. [86] found that patients in the probiotic (containing nine *Lactobacillus* and five *Bifidobacterium* species) group had higher levels of anti-inflammatoty and better clinical symptoms compared with the placebo group. Groeger et al. [87] demonstrated that *Bifidobacterium infantis* 35,624 achieved palliate effect to UC primarily by reducing intestinal inflammatory biomarkers (e.g. CRP, TNF-α, IL-6). In 2021, the study conducted by Gu et al. [88] revealed that *Akkermansia muciniphila* activate aryl hydrocarbon receptor (AhR) signaling, inhibite Kyn pathway (KP) activation, and restore the down-regulation of anti-inflammatory factors through increasing the levels of indoleacetic acid (IAA) and indole acrylic acid (IA) in the tryptophan (Trp) metabolic pathway. Similarly, the mitigation effect of probiotics (containing *L.casei* Zhang, *L.plantarum* P-8 and *B.animalis* subsp. *lactis* V9) was demonstrated in a trail by Chen et al. [89] in the treatment of UC. And the researchers found that the probiotic group had more beneficial bacteria, such as *Eubacterium ramulus*, *Pediococcus pentosaceus*, *Bacteroides fragilis* and *Weissella cibaria*.

All in all, these clinical studies have shown that the effectiveness of treating UC patients with probiotics is increasingly being proven. Above all, probiotics intervention might be a potentially effective approach in the treatment of UC by restoration of gut microbiota. Meanwhile, therapies that may most efficiently bring the disease under control are still being sought.

### Prebiotics therapy

**Fig. 3:**
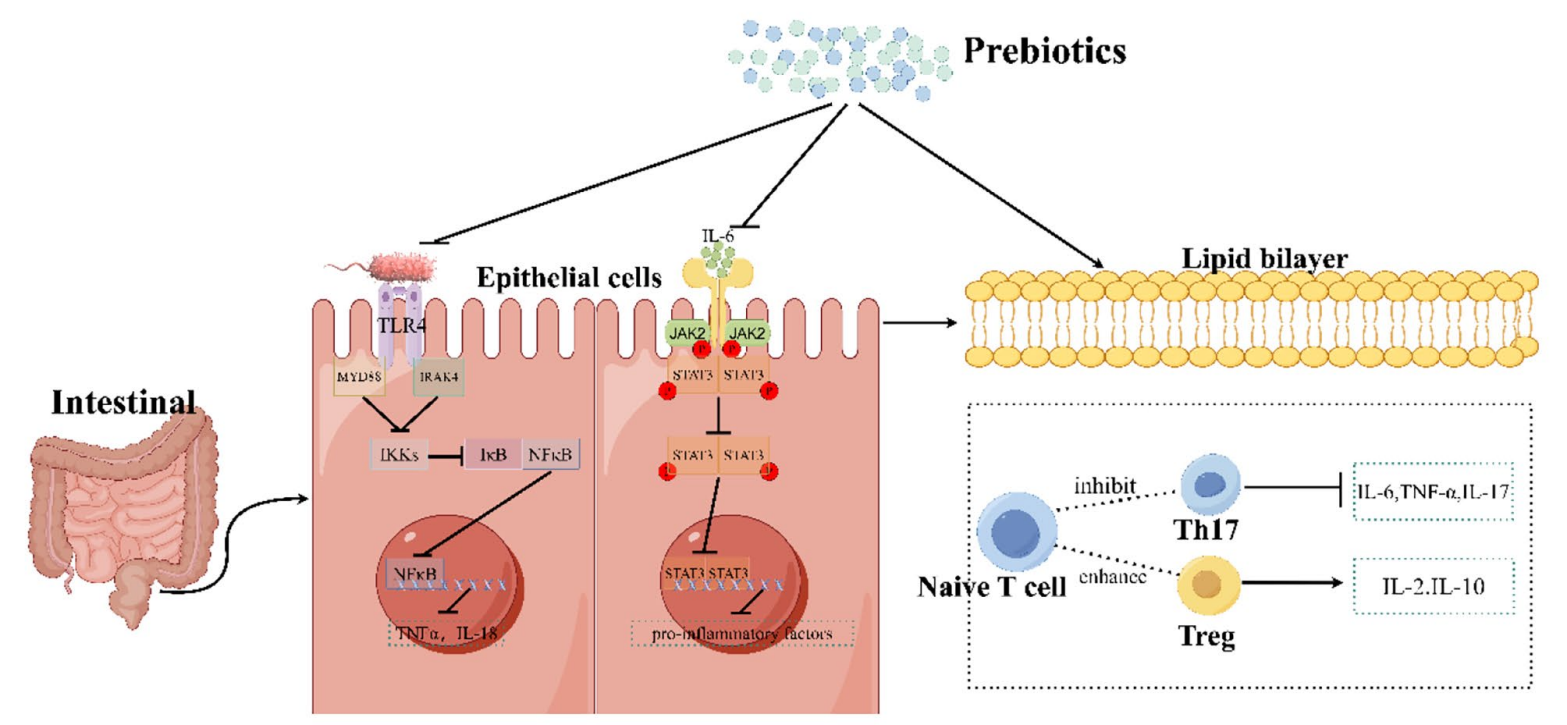
The mechanism of prebiotics in alleviating Ulcerative Colitis in Mice. It was found that the mechanism of prebiotics alleviate UC is probably through inhibiting of the TLR4/NF-κB signaling pathway, the JAK2/STAT3 signaling pathway, and regulating the ratio of T cell subsets. Firstly, prebiotics inhibit the activation effect of lipopolysaccharides from Gram-positive bacteria on TLR4 receptors, thereby inhibiting NF-κB from being released into nucleus and thus reducing the transcription of pro-inflammatory factors. Secondly, prebiotics can inhibit the activation of cytokine receptors by IL-6, thus suppress the entry of STAT3 into the nucleus and likewise inhibit its production of pro-inflammatory factors. Thirdly, prebiotics can inhibit of the conversion of naive T cells into Th17 cells and promote of their conversion into Treg cells, causing an increase of the expression of anti-inflammatory. (This mechanism diagram was drawn by Figdraw (https://www.figdraw.com))

Prebiotics are selectively fermentable, non-digestible oligosaccharides, or ingredients. They function to accelerate beneficial bacterial growth and suppress harmful bacterial growth, thus adjusting the balance of gut microbiota. In addition, they can lead to the production of SCFAs, regulate immune response, control gene expression in bacterial cells, and improve absorption of micronutrients. And prebiotics are used to treat a wide variety of disease, such as obesity [90], chronic enteritis [91], skin disease [92] and autism spectrum disorder [93]. The therapeutic effects of prebiotics on UC have also been confirmed in animal and humans (Tables 3 and 4). Thus, Prebiotics can be used as a novel dietary management approach for UC. Here, the possible effects and mechanisms of prebiotics in the treatment of UC are summarized in Fig. 3.

**Table 3:** Changes in microbiota composition in animals associated with UC and prebiotics therapeutic strategies

Models	Disease	New strategies	Implicated microbiota	Reference
Rat	UC	FOS	*Bifidobacterium* spp.↑, *Clostridium* cluster XI↓	[94]
Rat	UC	chicory-derived long-chain inulin-type fructans and short-chain inulin fraction oligofructose	*Bifidobacteria*↑, *Lactobacilli*↑	[95]
Mice	DSS-induced UC	stachyose	*Alistipes*↑, *Roseburia*↑, *Escherichia_Shigella*↓, *Parabacteroides*↓, *Romboutsia*↓, *Turicibacter*↓	[96]
Mice	UC	ENN IN	*Bifidobacterium* spp.↑, *Anaerostipes caccae*↑, *Escherichia Shigella* spp.↓	[97]
Mice	DSS-induced UC	HHPC and LHPC	Bacteroidetes↑, *Alloprevotella* genus↑, Firmicutes↓	[98]
Mice	DSS-induced UC	GBF	*Bifidobacterium*↑, *Eubacterium*↑	[99]

FOS, fructo-oligosaccharides; ENN IN, enriching exclusive enteral nutrition with inulin-type fructans; HHPC and LHPC, high-substituted hydroxypropyl cellulose and low-substituted hydroxypropyl cellulose; GBF, germinated barley foodstuff

**Table 4:** Changes in microbiota composition in human associated with UC and prebiotics therapeutic strategies

Models	Disease	New strategies	Implicated microbiota	Reference
Human	UC	Oligofructose and Inulin	bifibacteria↑, bacteroides↓, clostridia↓, fusobacteria↓	[100]
Human	UC	GOSs	bifidobacteria↑, lactobacilli↑, bacteroides↓, *C. histolyticum* group↓, *E. coli*↓, *Desulfovibrio* spp.↓	[101]
Human	UC	oligofructose-enriched inulin	No	[102]
Human	UC	GBF	*Bifidobacterium*↑, *Eubacterium limosum*↑, *Bacteroides*↓	[103, 104]
Human	UC	${2}^{{\prime }}$-FL	*Bifidobacteriu*↑, *Clostridium* cluster XIVa↑, *Roseburia* spp.↑, *Faecalibacterium prausnitzii*↑	[105]
Human	UC	BGS	*Bifidobacteria*↑	[106]
Human	UC	XOS	*Bifidobacteria*↑	[107]

GOSs, trans-galactooligosaccharide mixture; GBF, germinated barley foodstuff; ${2}^{{\prime }}$-FL, ${2}^{{\prime }}$-fucosyllactose; BGS, bifidogenic growth stimulator; XOS, Xylo-oligosaccharide

#### Experimental studies

Convincing evidence from animal studies indicate that prebiotics treatment can relieve UC. Koleva et al. [94] showed that fructo-oligosaccharides (FOS) promoted *Bifidobacterium* spp. and inulin and FOS can all decrease *Clostridium* cluster XI in rats, while *Bifidobacterium* spp. and *Clostridium* cluster XI correlated negatively and positively, respectively, to chronic intestinal inflammation. That is, both this two fructans inhibited intestinal inflammation. Hoentjen et al. [95] also orally administered a prebiotic combination of chicory-derived long-chain inulin-type fructans and short-chain inulin fraction oligofructose to HLA-B27 transgenic rats and found that this prebiotic can significantly reduce colitis and demonstrated that this effect was not only related to the gut microbiota, but also to immunomodulatory effects. They found that the prebiotic can promote the increase of bifidobacteria and endogenous lactobacilli. In immunomodulation, for example, it is possible to increase TGF-β in cecum. Wang et al. [96] allowed C57BL/6 mice with UC to receive oral administration of stachyose which is a prebiotic that traditionally extracted from plants for a period of time, and demonstrated the effect of stachyose on the recovery of body weight and found that it can reduced colonic tissue damage, lowered the level of pro-inflammatory cytokines, and restored the dysbiosis of the intestinal microbiota imblance (reduce the abundance of *Escherichia_Shigella*, *Parabacteroides*, *Romboutsia* and *Turicibacter* and raise the abundance of *Alistipes* and *Roseburia*). In the study of Lunken et al. [97], they used an adoptive T-cell transfer mice model of colitis to examine the effects of enriching exclusive enteral nutrition (EEN) with inulin-type fructans (IN) (ENN IN) on colitis and found that a less deterioration of the mucus layer, increased butyrate production, and the expansion of anti-inflammatory T-cell subsets, including IL-10 producing Foxp3^＋^ Tregs. And they also found an increased relative abundance of beneficial microbes (*Bifidobacterium* spp. and *Anaerostipes caccae*) and an reduced relative abundance of potentially pathogenic microbes (*Escherichia Shigella* spp.). All of these results continue to prove the benefits of prebiotics in UC. Li et al. [98] established the DSS-induced mice model of colitis by evaluating the therapeutic effects of prebiotics high-substituted hydroxypropyl cellulose (HHPC) and low-substituted hydroxypropyl cellulose (LHPC) on UC, and the results confirmed that these two prebiotics dose-dependently ameliorated the inflammation in colitis mice, inhibited pro-inflammatory cytokine and regulated the balance of intestinal flora, including increased the relative abundance of Bacteroides and Alloprevotella genus and reduced the relative abundance of Firmicutes. Kanauchi et al. [99] investigated the effect of Germinated barley foodstuff (GBF), a prebiotic product, on the gut environment and found that it can inhibited the expression of STAT3 and NF-κB, thereby reducing the inflammatory response of the epithelium.      

In summary, these animal experiments have showed the good effect of prebiotic therapy alone or in combination to UC. This provides a new direction in the clinical treatment of UC.

#### Clinical studies

Many clinical studies have demonstrated the benefits of prebiotics for people with UC. Oligofructose and Inulin as the oligosaccharide fraction of Raftilose and the oligosaccharide fraction of Raftiline, which was obtained by the extraction of chicory roots, Gibson et al. [100] have demonstrated the stimulatory effect of these two substances on intestinal bifibacteria, which is a bacterium thought to be beneficial to health through clinical experiment and reduced some pathogenic bacteria that can produce toxins or hydrolyzed proteins, including bacteroides, clostridia, and fusobacteria. Vulevic et al. [101] found that Galactooligosaccharides (GOSs) promoted the population of beneficial bacteria, especially bifidobacteria and lactobacilli, and reduced numbers of less beneficial bacteria (bacteroides, the *C. histolyticum* group, *E. coli*, and *Desulfovibrio* spp.), and also enhanced the immune response and reduced the production of pro-inflammatory factors. Similarly. Casellas et al. [102] demonstrated that oligofructose-enriched inulin reduced intestinal inflammation by measuring fecal calprotectin levels in patients. Faghfoori et al. [103] administrated germinated barley foodstuff (GBF) to patients with UC and showed that GBF were able to reduce serum levels of pro-inflammatory including IL-6, IL-8,TNF-α. As demonstrated by Mitsuyama et al. [104], by determining the changes of microorganisms in the feces of patients with UC after four weeks of oral administration of GBF, the results proved that prebiotics can increase the concentration of fecal *Bifidobacterium* and *Eubacterium limosum* and increase the concentration of colonic butyrate, which is a source of energy for epithelium. And decreased the presence of *Bacteroides*.

Ryan et al. [105] conducted in vitro and in vivo experiments and demonstrated the promoting effect of ${2}^{{\prime }}$-fucosyllactose (${2}^{{\prime }}$-FL) which is a prebiotic human milk oligosaccharide on butyric acid producers, including *Bifidobacterium*, *Clostridium* cluster XIVa and *Roseburia* spp. Butyric acid, on the other hand, as a kind of SCFA, can inhibit the inflammatory response. In this study, they also found a significant increase in fecal *Faecalibacterium prausnitzii*, *Anaerotruncus colihominis*, and *Pseudoflavonifractor* species. Consistent with the above results, Suzuki et al. [106] tested the effectiveness of Bifidogenic growth stimulator (BGS) which is a prebiotic preparation produced by Propionibacterium freudenreichii isolated from Swiss cheese in patients with UC and found that it can selectively stimulated the activation of *Bifidobacteria*, which not only produced butyrate to nourish colonocytes and inhibited cytokine production and activation of NF-κB pathway, but also improved the balance of the intestinal microflora to maintain intestinal mucosal integrity and prevented intestinal damage. In the clinical study by Li et al. [107], they demonstrated the potential of Xylo-oligosaccharide (XOS) to alleviate microecological dysbiosis in patients with UC by measuring the effect of XOS on the intestinal flora. They found that XOS promotes the proliferation of *Bifidobacteria*, which produces a variety of organic acids and inhibits the growth of harmful bacteria by altering their metabolites.

In conclusion, these clinical studies demonstrated the palliative effects of prebiotics on UC, showing that prebiotics hold promise as primary or adjunctive maintenance therapy for UC.

## Concluding remarks

UC as a common disease has become a financial burden for many people and has the potential to develop into cancer if not prevented or treated. Therefore, it is important to identify and intervene in a timely manner. The pathogenesis of UC is complex, that’s why it’s important to find a reliable treatment. There is a strong and complex relationship between gut microbiota and gut. Crucially, growing evidence strongly suggests that the gut microbiota plays a pivotal role in intestinal defense function, immune regulatory function, inflammatory responses, as a result, the development and progression of UC. Meanwhile, mechanistic studies have demonstrated these particular species of intestinal commensal bacteria capable of playing either a protective or pathogenic role in UC development. Traditional treatment methods come with a lot of side effects. And probiotics and prebiotics emerge as a new therapeutic modality to modulate the gut microbiota. Based on these, numerous animal and clinical studies have shown that regulating gut microbiota may be an effective strategy to treat UC.

Probiotics being able to confer notable health benefits by modulating the composition of gut microbiota and restoring the physiological bacterial flora. However, while an increasing number of studies have pointed to the therapeutic effects of probiotics on UC, the available data in this field remain limited and the relevant scientific work is still in its early stages. Thus, further research is still necessary. Firstly, due to the complex relationship between gut microbiota and UC, in order to better use probiotics to treat UC, it is necessary to further study the mechanism of intestinal flora affecting the occurrence and development of UC through more animal and clinical experiments. Secondly, we need to know how these probiotics regulate gut microbiota or how they function in the intestinal and what factors contribute to their long-run stability in both health and disease. Changes in certain pathway molecules can be probed to determine the specific mechanism of probiotic treats UC. Meanwhile, in the study of probiotics in the treatment of UC, we should pay more attention to the etiology and pathogenesis. Based on this, the composition and metabolites of probiotics should be of great concern. In particularly, it should be thoroughly studied for their antioxidant effects, anti-inflammatory properties, maintenance of the intestinal homeostasis, regulation of mucosal immune homeostasis, and so on. Some key probiotic components and metabolites may be highly effective postbiotic in the treatment of UC. Thirdly, most medications for the treatment of UC have many adverse effects. Meanwhile, probiotics have great potential as drugs to treat UC. Therefore, it may be more cost-efficient to invest more effort in probiotics than in developing new anti-inflammatory drugs. Fourth, in order to provide more effective probiotics to clinical, we can study the beneficial gut microbiota of healthy humans to dig out more and better probiotics. At the same time, it is necessary to search for the most effective probiotic compositions for the treatment of UC. Fifth, more clinical rationalized trials should be carried out to determine whether probiotics is safe and effective in the treatment of UC. Furthermore, because the composition of the gut microbiota is related to region, ethnicity, and diet, it is necessary to study large samples of people in different regions. Sixth, we must figure out the route of administration of the probiotics as well as the dosage, to ensure the probiotics will maximize the benefits in patient’s body under safe administration. Seventh, in order to make it easier and more convenient for patients to use probiotics, such as how to keep probiotics maintain highly active in some way and make it easier for patients to take, we should further explore the production and preservation of probiotics. Last but not least, to accepted by patients as a reliable treatment, it should be clarified for which patients a particular probiotic is effective, or which is preferable for a single probiotic or a blend of strains. So, there are still many problems it faces. In the future, probiotic therapy may be a potentially useful approach for UC, but research in this area has just started.

Prebiotics offer an exciting new approach to dietary management of gastrointestinal disorders including UC. It has been accepted as a dietary food ingredient that helps to nourish gut microbes, which can improve health and prevent UC. But while many studies to date have demonstrated the beneficial effects of prebiotics in UC, it still faces numerous challenges. Now many studies have the limitation of too small a sample size or lack of a control group, so the evidence for a significant effect of prebiotics is still lacking. The dosage of prebiotics is also a question to be confirmed, if too high a dosage will lead to tolerance, or if a higher dosage of prebiotics will produce better results when well tolerated. With so many types of prebiotics available, it is also deserving of further study as to which prebiotics have better results for which type of UC patients. Although a large number of in vitro and in vivo experiments have confirmed the positive effects of prebiotics, there is still a need for more clinical trials or animal experiments to further evaluate their specific effects.The specific mechanism by which we found that prebiotics alleviate UC remains unclear. It’s worth exploring further. In-depth experiments are needed to further elucidate the role of prebiotics in patients with UC, whether it is their own structure or their metabolites that play a role. And to meet the needs of consumers, new strategies for cost-effective and efficient prebiotics can be developed. Prebiotics, as a food-sourced ingredient for the treatment of UC, offer a new clinical direction, and it is important to study its good effects and side effects as clearly as possible. Therefore, in any case, the prospect of the application of prebiotics in UC is worthy of attention and expectation.

Certainly, in order to gain wider acceptance and recognition for probiotics and prebiotics to treat UC, further research is urgently required.

## Data Availability

The data that support the findings of this study are available from the corresponding author, upon reasonable request.
