# Correction: The development of probiotics and prebiotics therapy to ulcerative colitis: a therapy that has gained considerable momentum

**DOI:** 10.1186/s12964-024-01674-y

**Published:** 2024-05-27

**Authors:** Jing Guo, Liping Li, Yue Cai, Yongbo Kang

**Affiliations:** 1 Department of microbiology and immunology, School of Basic Medical Sciences, Shanxi Medical University, Taiyuan, Shanxi China; 2 Faculty of Life science and Technology, Kunming University of Science and Technology, Kunming, Yunnan China

**Correction: Cell Commun Signal 22, 268 (2024)**

10.1186/s12964-024-01611-z

Following publication of the original article [1], the authors reported an error in the corresponding authorship of the article. Yongbo Kang is the sole corresponding author.

The Author contributions statement also included the incorrect information.

The incorrect statement is:

Jing Guo wrote the main manuscript text with the help of Liping Li, Xinwei Huang and Yongbo Kang. All authors reviewed the manuscript.

The corrected statement should read as follows:

Jing Guo wrote the main manuscript text with the help of Liping Li, Yue Cai and Yongbo Kang. All authors reviewed the manuscript.

The original article [1] has been corrected.
